# Intranasally inoculated bacterium-like particles displaying porcine epidemic diarrhea virus S1 protein induced intestinal mucosal immune response in mice

**DOI:** 10.3389/fimmu.2023.1269409

**Published:** 2023-09-18

**Authors:** Kai Su, Yawen Wang, Chen Yuan, Yanan Zhang, Yanrui Li, Tanqing Li, Qinye Song

**Affiliations:** ^1^ College of Veterinary Medicine, Hebei Agricultural University, Baoding, Hebei, China; ^2^ National Center of Technology Innovation for Pigs, Chongqing, China; ^3^ Hebei Veterinary Biotechnology Innovation Center, Baoding, Hebei, China

**Keywords:** PEDV, S1 protein, bacterium-like particles, immunization routes, mucosal immunity

## Abstract

Porcine epidemic diarrhea virus (PEDV) causes acute watery diarrhea and high mortality in newborn piglets. Activation of intestinal mucosal immunity is crucial to anti-PEDV infection. To develop a vaccine capable of stimulating intestinal mucosal immunity, we prepared a bacterium (*Lactococcus lactis*)-like particle (BLP) vaccine (S1-BLPs) displaying the S1 protein, a domain of PEDV spike protein (S), based on gram-positive enhancer matrix (GEM) particle display technology. We further compared the effects of different vaccination routes on mucosal immune responses in mice induced by S1-BLPs. The specific IgG titer in serum of intramuscularly immunized mice with S1-BLPs was significantly higher than that of the intranasally administered. The specific IgA antibody was found in the serum and intestinal lavage fluid of mice vaccinated intranasally, but not intramuscularly. Moreover, the intranasally inoculated S1-BLPs induced higher levels of IFN-γ and IL-4 in serum than the intramuscularly inoculated. In addition, the ratio of serum IgG2a/IgG1 of mice inoculated intramuscularly was significantly higher with S1-BLPs compared to that of with S1 protein, suggesting that the immune responses induced by S1-BLPs was characterized by helper T (Th) cell type 1 immunity. The results indicated that S1-BLPs induced systemic and local immunity, and the immunization routes significantly affected the specific antibody classes and Th immune response types. The intranasally administered S1-BLPs could effectively stimulate intestinal mucosal specific secretory IgA response. S1-BLPs have the potential to be developed as PEDV mucosal vaccine.

## 1 Introduction

Porcine epidemic diarrhea virus (PEDV), a highly contagious enterovirus, causes acute diarrhea, vomiting, dehydration and death in pigs, causing huge economic losses to the swine industry worldwide (1, 2). PEDV infects pigs of all ages, but neonatal piglets under 7 days old are more susceptible to PEDV infections, with a mortality of up to 100% (2, 3). In addition to direct and indirect fecal-oral routes, it has been confirmed that PEDV can be transmitted to the intestinal epithelium through the respiratory route (4). PEDV belongs to the genus *Alphacoronavirus* in the family *Coronaviridae* and is an enveloped single-stranded positive-sense RNA virus, with a genome size of about 28 k (5). Spike glycoprotein (S), composed of 1383-1386 amino acids (aa), is a type I membrane protein consisting of S1 and S2 subunits on the viral surface as a trimer. During viral infection, the N-terminal S1 subunit (1-789 aa) is responsible for receptor binding, and the C-terminal S2 subunit (790-1383 aa) is involved in the fusion of the viral envelope with the host cell membrane (6, 7). The S1 subunit is an important determinant of the virulence of PEDV and a major target of neutralizing antibodies (8–11). Therefore, subunit vaccines based on full-length or truncated S1 protein can effectively elicit protective antibody responses *in vivo* (12–15).

In general, although PEDV can also cause transient viremia in young piglets, it mainly causes localized intestinal infections. This phenomenon requires new vaccination strategies that focus on the induction of mucosal immunity to protect the intestinal mucosa. Moreover, due to the high susceptibility and immaturity of the immune system in neonatal piglets, passive lactogenic immunity to PEDV is critical for suckling piglets to obtain protection. IgA titers in colostrum are correlated with PEDV-neutralizing antibody titers (16, 17). Therefore, increasing the specific secretory IgA (sIgA) titers in colostrum via maternal immunity is the most effective strategy to protect newborn piglets against PEDV (18, 19). To date, attenuated or inactivated PEDV vaccines have been widely used (20, 21). However, existing vaccines are not so effective that some vaccinated sows or gilts do not develop protective lactogenic immunity for the neonatal. Meanwhile, there are some difficulties in the cultivation of PEDV, resulting in high production costs of attenuated or inactivated PEDV vaccines. Existing vaccines also have potential biosafety risks. Though vaccination via the traditional route such as intramuscular injection is effective in inducing systemic immune responses, it is difficult to elicit antigen-specific mucosal immune responses (22). Additionally, the triggering of immune responses is closely correlated with the nature of the antigen and vaccination routes. Even the same antigen with different vaccination routes causes different immune response types. Therefore, it is of practical significance to explore new vaccines and vaccination routes that can induce mucosal immune responses for the prevention and control of PEDV.

The heterologous display of proteins or peptides on the surface of microorganisms is an emerging and interesting technology with wide applications in various fields. Heat-killed non-recombinant lactic acid bacteria (LAB) or non-viable bacterium-like particles (BLPs) obtained by the pretreatment of whole bacteria in hot acid are designated as Gram-positive enhancer matrix (GEM) particles, which consisted mainly of bacterium-derived peptidoglycan spheres without other intact cell wall components and intracellular components (23). The GEM particles provide a suitable cell surface that can display various heterologous proteins through a peptidoglycan-binding domain, i.e., protein anchor (PA). The PA is derived from the *Lactococcus lactis* peptidoglycan hydrolase AcmA and contains three LysM motifs consisting of about 45 amino acids separated by spacer sequences, which can specifically bind to GEM particles and enable the display of heterologous proteins on their surface (24). Therefore, the GEM-PA is not only a safe, attractive and affordable antigenic surface display system but also a mucosal vaccine delivery system. BLPs can improve the systemic immune responses and local mucosal immune responses in animals through intranasal, oral and intramuscular injection immunization routes (25–27).

**Figure 1:**
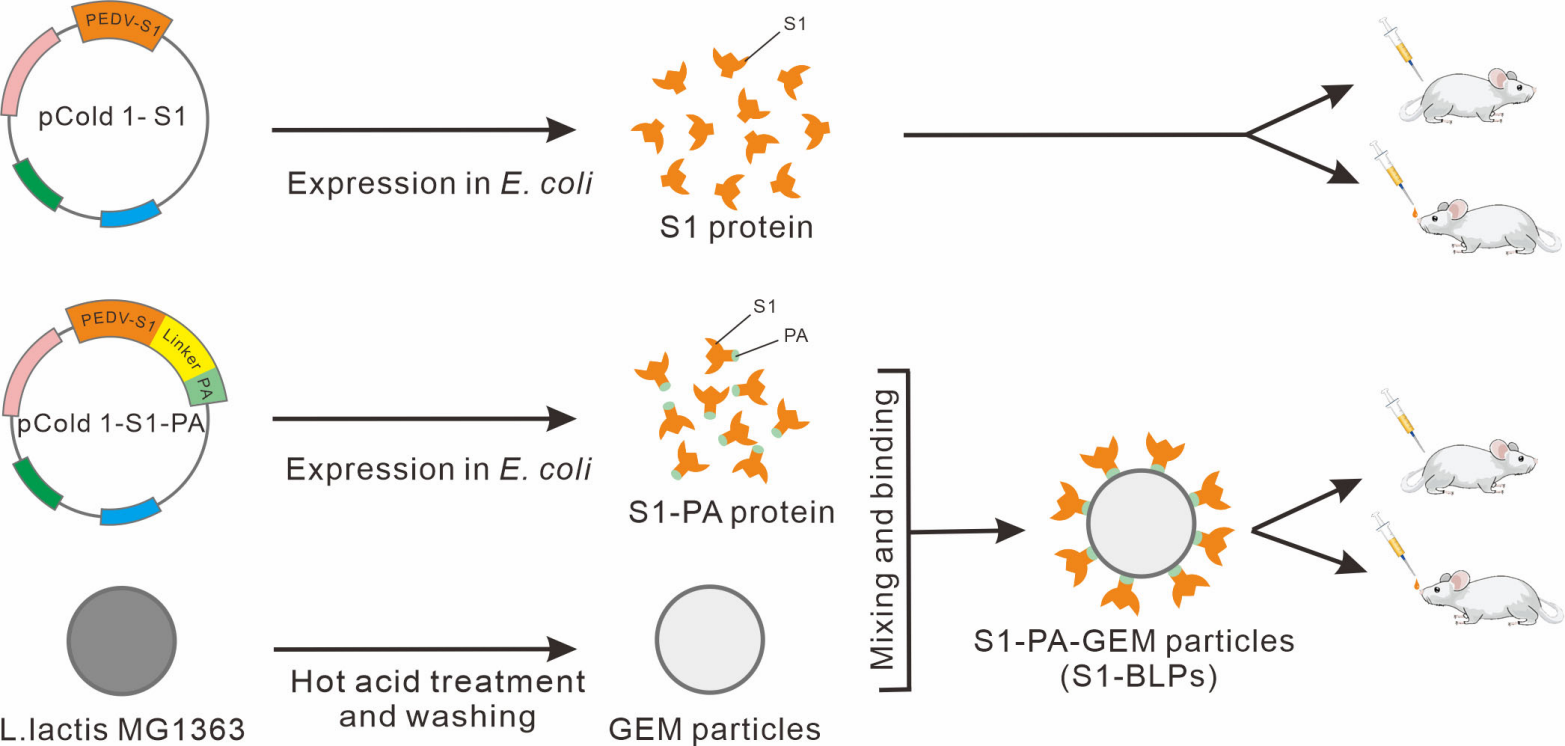
Schematic diagram of S1 protein, S1-PA-GEM particle preparation, and animal immunization. The pCold 1-S1 refers to S1 protein recombinant expression plasmids, and pCold 1-S1-PA refers to expression plasmids of recombinant protein (S1-PA) of S1 protein and the protein anchor (PA), containing a flexible linker sequence between S1 gene and PA gene sequences. The recombinant protein S1-PA and the gram-positive enhancer matrix (GEM) particles were used to prepare S1-BLPs for intramuscular or intranasal immunization of BALB/c mice.

To develop a safe PEDV vaccine that can induce robust immune responses in the intestinal mucosa, S1-PA fusion protein was expressed by *Escherichia coli* (*E. coli*) in this study, and S1-BLPs displaying S1 protein was prepared using the strategy described in **Figure 1**. Mice were immunized with S1-BLPs by either intramuscular injection or intranasal administration, and we compared the differences in specific local mucosal and systemic immune responses induced by S1-BLPs between two immunization routes.

## 2 Materials and methods

### 2.1 Construction of recombinant plasmids

The PEDV S1 gene was amplified from the genome of PEDV QY-2016 strain (GenBank ID: MH244927) preserved in our laboratory by PCR using primers S1-F and S1-R (**Table 1**). The DNA sequence of the PA gene based on the peptidoglycan hydrolase AcmA (GenBank ID: U17696) was synthesized by Sangon Biotech (Shanghai, China). The S1-PA fusion protein gene *S1-PA* was obtained using overlap extension PCR (OE-PCR) by splicing the segments of S1 gene and PA gene through a flexible linker (GGSG). All the primers in this study were synthesized by Sangon Biotech (Shanghai, China). To obtain the recombinant expression plasmid pCold1-S1 or pCold1-S1-PA, the gene *S1* and the fusion protein gene *S1-PA* were digested with *EcoR* I and *Sal* I, respectively, and then cloned into the pCold 1 vector carrying a 6×his tag (Takara Bio, #3360) also digested with the above restriction endonucleases using T4 ligase. Finally, the single clone carrying *S1* or *S1-PA* gene was verified by PCR and double digestion with *EcoR* I and *Sal* I, and then was sequenced by Sangon Biotech (Shanghai).

**Table 1:** Primers and corresponding sequences used in this study.

Primer	Sequence (5′→3′)	Restriction enzyme	Description	Annealing temperature/°C
S1-F^a^	TCA*GAATTC*ATGGTACTCGGCGGTTATCTA	*Eco*R I	S1(2244bp) and S1-linker-PA(2943bp)	56 °C
S1-R	TGT*GTCGAC*TTAACTAAAGTTGGTGGGAAT	*Sal* I	S1(2244bp)	56 °C
S1-linker-R^b^	ACCACCACCAGAACCACCACTAAAGTTGGTGGGAATA		Overlapping Extension PCR for S1-linker-PA(2943bp)	56 °C
linker-PA-F^c^	GGTGGTTCTGGTGGTGGTTCTGGTGACGGAGCTTCTTCAG		Overlapping Extension PCR for S1-linker-PA (2943bp)	53 °C
PA-R^d^	ACGC*GTCGACT*TATTTTATTCGTAGATAC	*Sal* I	PCR for PA and S1-linker-PA	53 °C

^“a” and “d”^: Italics indicate restriction sites.

^“b” and “c”^: Underlined letters indicate the gene sequence of linker.

### 2.2 Expression and purification of the protein

The recombinant plasmids pCold1-S1 and pCold1-S1-PA were transformed into competent *E. coli* BL21 (DE3) cells (Tiangen Biotech, #CB105) using the heat shock method. The transformed *E. coli* cells were inoculated on Luria Broth (Amp^+^/LB) Agar plates containing 50 μg/mL ampicillin and cultured at 37°C for 18 h. The single colony containing the target gene was selected and inoculated into Amp^+^/LB broth for culture at 37°C. When the OD_600 nm_ value of the bacterial culture reached 0.6-0.8, the culture was moved to 4°C for 1 h. After adding 0.5 mmol/L isopropyl-β-d-thiogalactoside (IPTG), the bacteria were cultured at 16°C for 24 h to induce the expression of recombinant proteins. The bacterial cells were harvested and re-suspended in PBS (pH 7.4), followed by sonication in an ice bath, the supernatant was collected for SDS-PAGE analysis after centrifugation at 10,000 r/min for 10 min. The expressed proteins were purified using the HisTrap™ HP (GE company, #17524801) on the AKTA protein purification system (GE company, USA), and confirmed by Western blotting. The protein concentration was measured using the BCA protein assay kit (Takara Bio, #T9300A).

### 2.3 Western blotting

SDS-PAGE was used to analyze the expression and purification of S1 and S1-PA proteins, as well as the proteins bound to BLPs. S1 protein, S1-PA and BLPs-bound S1 protein were identified by Western blotting. Briefly, the recombinant protein was transferred to a polyvinylidene difluoride (PVDF) membrane following SDS-PAGE, and blocked overnight at 4°C. The membrane was incubated with anti-His-tagged mouse monoclonal antibody (1:5000; Cowin Biotech, #CW0285) or Rabbit anti-S1 protein polyclonal antibody (1:500; prepared and stored in our lab) at room temperature (RT) for 1 h. After washing, the membrane was incubated with 1:5000 diluted HRP-conjugated goat anti-mouse/-rabbit antibody (Solarbio Tech, #SE131, #SE134) at RT for 1 h. After washing, the membrane was incubated in the DAB chromogenic solution for color development, and the results were observed directly.

### 2.4 Preparation of S1-BLPs

*Lactococcus (L.) lactis* MG1363 was cultured in GM17 broth (Hope Bio, #HB0391) at 30°C for 24 h with shaking. Bacterial cells were harvested by centrifugation at 8,000 r/min for 15 min, washed twice with sterile PBS, re-suspended in 25 mM sulfuric acid, and heated at 100°C for 30 min. The treated *L. lactis* (GEM) particles were centrifuged, and the pellet was washed three times with PBS. Finally, the GEM particles (GEMs) were re-suspended in PBS at a concentration of 2.5×10^9^ particles/mL, which was referred to as 1 U (28). The GEM particles were directly used for the preparation of S1-BLPs or stored at -20°C.

To prepare S1-BLPs, the GEM particles (2.5×10^9^ particles/mL) were mixed with 6.25 mg of the fusion protein S1-PA followed by incubation at RT for 30 min with shaking. The GEM particles bound to S1 protein via PA, designated S1-BLPs, were collected by centrifugation at 6,000 r/min for 5 min, washed 3 times with PBS, and re-suspended in PBS. To confirm the binding of the fusion protein S1-PA to the GEM particles, S1-BLPs was treated with 10% SDS at 100°C for 10 min to observe whether S1-PA was dissociated from the GEM particles. GEM particles control was set up at the same time. After centrifugation at 10,000 r/min for 2 min, the supernatant was collected and analyzed by SDS-PAGE to confirm the presence of S1-PA. Meanwhile, BCA protein assay kit was used to determine the total S1-PA protein concentration in the supernatant, and using the following formula to calculate the amount of protein bound by GEMs per unit (2.5×10^9^ particles/mL). Amounts of GEMs binding S1-PA protein per unit (μg) = (Total protein amounts in per unit of S1-BLP supernatant) - (Total protein amounts in per unit of GEM supernatant).

### 2.5 Transmission electron microscopy

The samples were dropped onto the copper grid, negatively stained with 2% phosphotungstic acid, and vacuum dried. The samples were examined on a transmission electron microscope (TEM) (JEM1400, JEOL, Tokyo, Japan), which was operated at 80 kV and equipped with An AMT Camera. The particle sizes were measured using Image J.

### 2.6 Indirect immunofluorescence assay

S1-BLPs was evenly spread on the polylysine coated slides, air-dried, and blocked with 3% BSA in PBS at RT for 30 min. After washing twice with PBS, the slides were incubated with anti-PEDV S1 protein rabbit polyclonal antibody (1:100) at RT for 60 min. Meanwhile, the anti-S1 protein antibody negative rabbit serum control was set up. After washing 3 times with PBS, the slides were incubated with FITC-labeled goat anti-rabbit IgG (1:200, Solarbio Tech, # SF134) for 60 min at RT in the dark. The slides were washed three times with PBS, and the fluorescence was observed under an Axio Observer D1 fluorescence microscope (ZEISS, Gottingen, Germany).

### 2.7 Immunization and sample collection

The S1 protein or S1-BLPs were mixed with an equal volume of Montanide™ IMS1313N VG (IMS1313 for short) water adjuvant (Seppic, Paris, France). Forty specific-pathogen-free (SPF) female BALB/c mice (Changsheng Biotech, Liaoning, China) aged 6-8 weeks were randomly divided into 5 groups (S1/IM; S1-BLPs/IM, S1/IN, S1-BLPs/IN, and Blank group) with 8 mice in each group. Each mouse in S1/IM and S1/IN groups was immunized three times by intramuscular (IM) and intranasal inoculation (IN) with a dose 80 μL contained 40 μg of S1 protein at 2-week intervals, respectively. And each one in BLPs IM and BLPs IN groups was immunized with S1-BLPs containing 40 μg S1 protein by the same routes as above, respectively. In the blank group, mice were not immunization. Blood samples were collected from the tail vein of mice weekly before and after immunization, and serum collection was conducted and stored at -20°C for subsequent tests. At 14 days after the second immunization and 21 days after the third immunization, 4 mice were randomly selected from each group and anesthetized by intraperitoneal injection of 10% chloral hydrate (0.1 mL/10g body weight), and then intestine and lung airway lavage fluid were collected for specific secretory IgA detection.

### 2.8 Detection of specific antibodies

PEDV S1-specific IgG, IgA, IgG1, IgG2a and IgG2b antibodies in serum were measured by ELISA, and IgG titer was determined on day 21 after the third immunization. Moreover, the ratio of IgG2a/IgG1 in serum of S1/IM and S1-BLPs/IM groups was analyzed. Briefly, 96-well ELISA plates (Biofil, #FEP101896) were coated with PEDV S1 protein (2 μg/well) diluted with Coating buffer (0.1 M carbonate buffer, pH 9.0) at 37°C for 1 h and overnight at 4°C. The plates were blocked with Blocking buffer (5% skim milk powder in PBST) at 37°C for 1 h. After washing 3 times with PBST (0.05% Tween-20 in phosphate-buffered saline (PBS), pH 7.4), 100 μL of samples (serum, or intestine or lung airway lavage fluid), or 4-fold serially diluted (1:100 to 1:204 800) serum were added in the wells of the plate to incubate at 37°C for 60 min. At the same time, positive, negative, and blank controls were set up. After washing 3 times, 100 μL of HRP-conjugated goat anti-mouse IgG (1:15000, Biodragon Tech, #BF03001) or IgA/IgG1/IgG2a/IgG2b antibodies (1:1000, Biodragon Tech, #BF03007, #BF03050, #BF03051, #BF03052) were added at 37°C for 60 min. After washing 3 times, TMB single-component substrate solution (Solarbio Tech, #PR1200) was added, 100 μL/well, to incubate in the dark at RT for 15 min. Finally, 50 μL stop solution was added to each well to terminate the reaction, and the OD_450_ nm value was measured using a Multimode Microplate reader (Biotek Synergy HTX, USA). Three parallel repeated tests were performed for each sample.

### 2.9 Detection of cytokine

The levels of IFN-γ, IL-2 and IL-4 in serum were detected using mouse cytokine ELISA kits (Shanghai Enzyme-linked Biotech, #m1002277, #ml063136, #ml063156) on day 21 after the third immunization. Briefly, 50 μL of serum diluted 1:3 and 100 μL of HRP-labeled specific antibody were added into each well followed by incubation at 37°C for 60 min. After washing 3 times with PBST, the substrate solution was added into each well to develop in the dark at 37°C for 15 min. The reaction was stopped immediately with the stop solution, 50 μL/well, and the OD values were measured at 450 nm using a Multimode Microplate reader (Biotek Synergy HTX, USA). Three parallel repeated tests were performed for each sample.

### 2.10 Statistical analysis

All data presented in this study were expressed as mean ± standard deviation (SD) and analyzed using GraphPad Prism 8.0. Statistical analysis was conducted using One-way ANOVA, followed by Duncan’s multiple comparisons. Differences among different groups were considered significant when *P* < 0.05 (*) or *P* < 0.01 (*), and the comparison without a statistics bar and asterisk were found to be non-significant (*P* > 0.05).

## 3 Results

### 3.1 Identification of recombinant expression plasmids

The constructed recombinant expression plasmids, pCold 1-S1 and pCold 1-S1-PA, were identified by PCR, the restriction endonuclease (*EcoR* I and *Sal* I) digestion, and sequencing and analysis, respectively. After agarose gel electrophoresis, target bands of the same size, 2244 bp and 2943 bp, as the expected S1 and the S1-PA recombinant protein was observed, respectively (**Supplementary Figure 1**). The sequencing results were consistent with the reference sequences.

### 3.2 Expression of recombinant protein and identification by Western blotting

**Figure 2:**
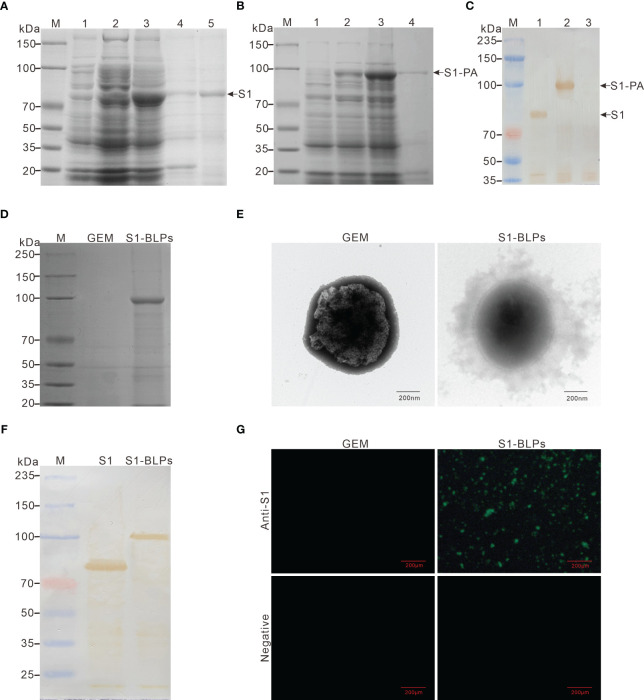
Expression of recombinant proteins and preparation of S1-BLPs. **(A–C)** Identification of expressed recombinant protein. SDS-PAGE analysis of recombinant proteins S1 **(A)** and S1-PA **(B)**, respectively. M: Protein marker; 1 and 2: *E. coli* BL21 (DE3) before and after induction with IPTG, respectively; 3: Lysate supernatant of induced *E. coli* BL21 (DE3); 4: Lysate precipitate of induced *E*. *coli* BL21 (DE3); lane 5: Purified S1 protein. **(C)** Identification of recombinant proteins S1 and S1-PA by Western blotting. M: Protein marker; 1: Protein S1; 2: Protein S1-PA; 3: Negative control. **(D)** SDS-PAGE analysis of S1-PA recombinant protein on the surface of GEM. **(E)** Morphology of S1-BLPs under the transmission electron microscopy. **(F)** Identification of S1 protein on S1-BLPs by Western blotting. S1 (the control) or S1-BLPs reacted with Rabbit anti-S1 protein polyclonal antibody. **(G)** Indirect immunofluorescence assay. GEMs and S1-BLPs reacted with Rabbit anti-S1 protein polyclonal antibody or the anti-S1 protein antibody negative rabbit serum, respectively.

SDS-PAGE analysis and Western blotting showed the recombinant proteins of S1 and S1-PA were successfully expressed in the *E. coli* BL21 (DE3) cells by IPTG-induced 24 h at 16°C (**Figures 2A–C**). The expressed proteins existed mainly in the supernatant of the bacterial lysate at 80 kDa and 104 kDa, respectively. Recombinant S1 protein was purified by metal affinity chromatography, and after desalination and concentration, the protein concentration was 2.4-3.2 mg/mL. S1-PA recombinant protein was directly used for the subsequent preparation of S1-BLPs.

### 3.3 Display of S1 protein on GEMs surface via PA

After incubation of GEM particles with the S1-PA protein, S1 antigen could be displayed on GEMs surface by anchoring protein PA. When S1-BLPs was subjected to SDS-PAGE electrophoresis, S1-PA recombinant protein bands were seen in the corresponding lanes (**Figure 2D**). BCA protein assay showed that each unit (2.5×10^9^ particles/mL) of the GEMs could bind to 371-410 μg of S1-PA protein. Furthermore, the electron microscopy revealed a ring of flocculation around the GEMs in S1-BLPs with a diameter of about 800-1000 nm, but not around the GEMs that did not bind the S1 protein (**Figure 2E**). These results showed that S1 protein was conjugated with GEMs and displayed on the surface of GEMs.

### 3.4 Identification of S1 protein on S1-BLPs

The antigens in S1-BLPs were detected by Western blotting and immunofluorescence assay. As shown in **Figure 2F**, a clear brown band of around 104 kDa appeared on the PVDF membrane in the lane of S1-BLPs following Western blotting. As expected, S1-BLPs glowed green fluorescence after indirect immunofluorescence staining while GEMs control not (**Figure 2G**). The results demonstrated that S1 protein was conjugated with GEMs by the protein anchor.

### 3.5 Specific IgG and IgG subclasses in serum

**Figure 3:**
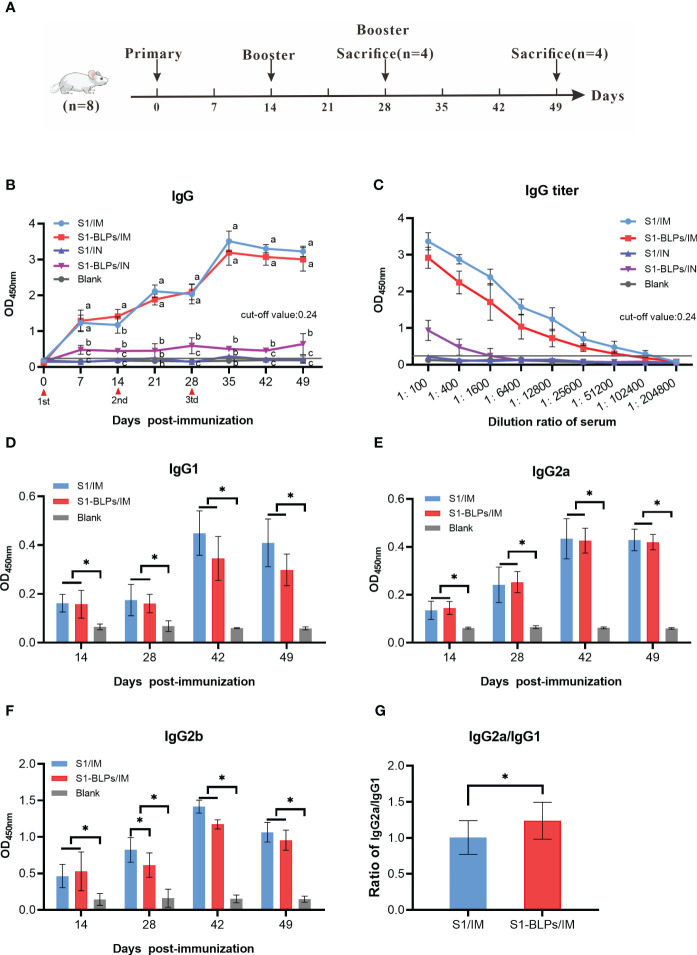
Timeline of the mouse immunization and specific IgG and IgG subclasses in serum. **(A)** Timeline of experimental treatment of mice. 40 female BALB/c mice aged 6-8 weeks were randomly divided into 5 groups with 8 mice in each group. Before and after immunization, blood samples were collected from the tail vein of mice weekly. At 14 days after the second immunization and 21 days after the third immunization, 4 mice were randomly selected from each group for the collection of intestine and lung airway lavage fluid, respectively. **(B)** Dynamics of specific IgG. **(C)** Specific IgG titers on day 21 after the third immunization. **(D–F)** Specific IgG1, IgG2a, IgG2b antibodies in serum. **(G)** Ratios of IgG2a to IgG1 at 14, 28, 42, and 49 days post immunization. The data came from three parallel replicates of each sample. Bars show means ± SD. Lowercase or *P<0.05.

In order to analyze the influences of immunization routes on immune responses and compare systematic specific IgG antibody induced by S1-BLPs and S1 protein, female BALB/c mice were immunized with S1-BLPs or S1 protein through the intramuscular and intranasal route, respectively, and serum was collected weekly (**Figure 3A**). After immunization, the specific IgG antibody levels in two intramuscular immunization groups (S1/IM and S1-BLPs/IM) were significantly higher than those in two intranasal inoculation groups (S1/IN and S1-BLPs/IN) (*P*<0.05) (**Figure 3B**). Although the IgG antibody in S1-BLPs intranasal immunization (S1-BLPs/IN) group remained at a relatively low level, it higher than that in S1/IN group. At 14 days after the primary immunization, the IgG level in S1-BLPs/IM group was significantly higher than that in S1/IM group (*P*<0.05), but there were no significant differences after the second and third immunizations (*P*>0.05). No specific IgG antibody was found in S1/IN and the blank group. The antibody titers in groups S1/IM and S1-BLPs/IM reached 1:102400 and 1:51200, respectively on day 21 after the third immunization, which were significantly higher than 1:1600 in the intranasal S1-BLPs group (**Figure 3C**), indicating that the intranasal immunization of S1-BLPs has limited ability to stimulate systemic specific IgG antibodies.

The levels of specific IgG subclasses in serum were also tested by ELISA. Due to the low total IgG levels in the intranasal immunization groups, only IgG subclasses of the intramuscular immunization groups were measured. As shown in **Figure 3D**, the IgG1 antibody levels in the S1/IM group was slightly higher than that in the S1-BLPs/IM group at 14 and 28 days after the third immunization, but there was no significant difference between them (*P*>0.05). S1/IM group and S1-BLPs/IM group had similar IgG2a levels (*P*>0.05), and the former exhibited a higher IgG2b antibody level on day 14 after the second immunization (*P*<0.05) (**Figures 3E, F**). Moreover, S1-BLPs/IM group had a higher ratio of IgG2a/IgG1compared to the S1/IM group (*P*<0.05) (**Figure 3G**), indicating that the immune response induced by S1-BLPs was characterized by T helper (Th) cell type 1 (Th1) immunity.

### 3.6 Specific IgA in serum and sIgA in intestine and bronchoalveolar lavage fluid

**Figure 4:**
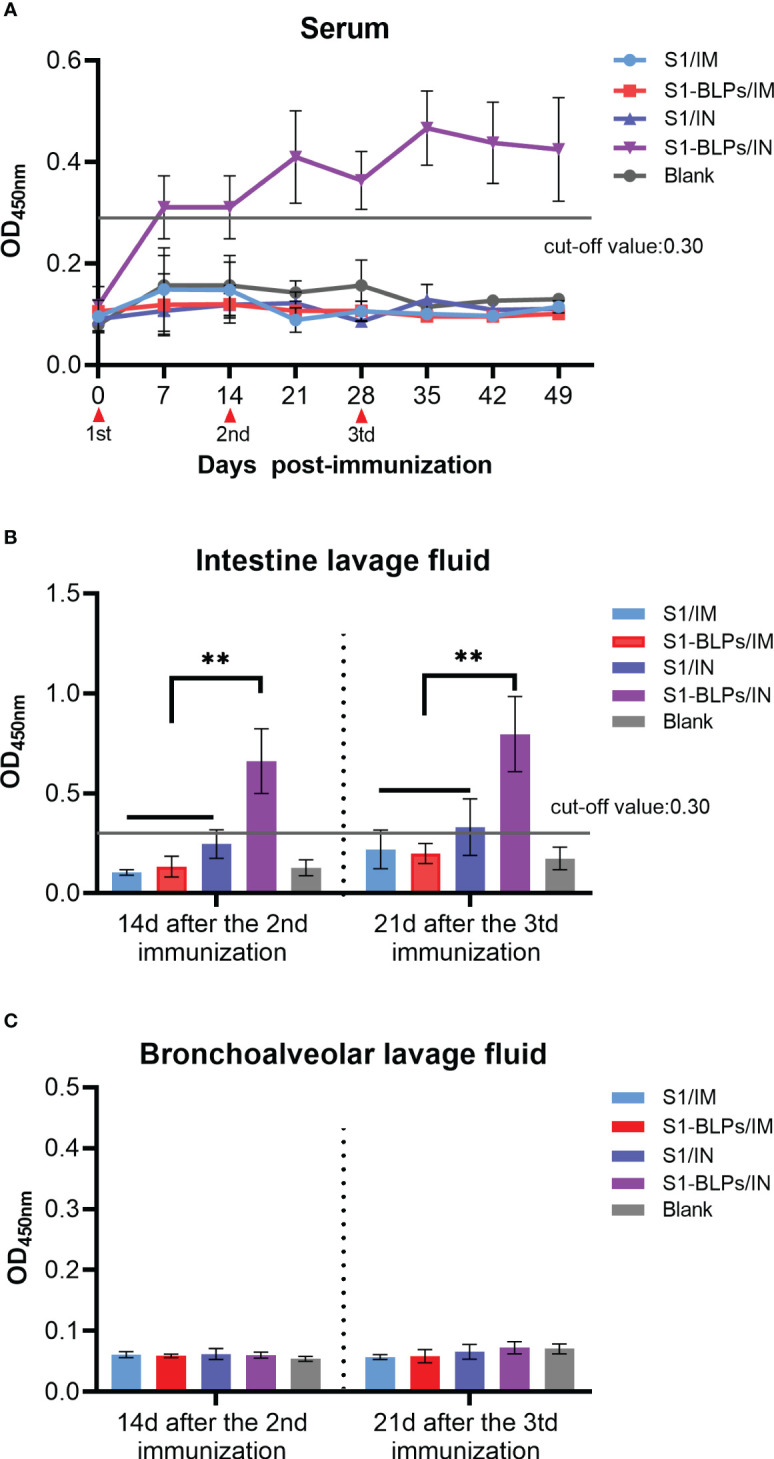
IgA in serum and secretory IgA in lavage fluid by ELISA. When the OD value is >0.3, the specific IgA is positive, and when it <0.3, the specific IgA is negative. **(A)** Dynamics of specific IgA in serum. **(B)** Specific secretory IgA in small intestinal lavage fluid. **(C)** Specific secretory IgA levels in bronchoalveolar lavage fluid. The data came from three replicates of each sample. Bars show mean ± SD. **P<0.01. The data came from three parallel replicates of each sample.

To evaluate the effects of immunization routes on the specific IgA and sIgA responses, we compared the levels of IgA in serum and sIgA in the intestine and bronchoalveolar lavage fluid. From 7 days after immunization until the end of the experiment, only seroconversion of IgA presented in S1-BLPs/IN group (*P*<0.05). There was no significant difference between groups S1/IM, S1-BLPs/IM or S1/IN and the blank group (*P*>0.05) (**Figure 4A**). At 14 days after the second immunization and 21 days after the third immunization, the OD values of specific sIgA in intestine lavage fluid were 0.246 and 0.330 in S1/IN group, and 0.660 and 0.796 in S1-BLPs/IN group, respectively (**Figure 4B**). There was a significant difference between the two groups (*P*<0.01). No specific sIgA was found in the other groups (**Figure 4B**). In bronchoalveolar lavage fluid of all groups, specific sIgA was not detected (**Figure 4C**). The result showed that S1-BLPs could induce systemic and intestinal mucosal specific IgA responses by intranasal immunization.

### 3.7 Cytokines in serum

**Figure 5:**
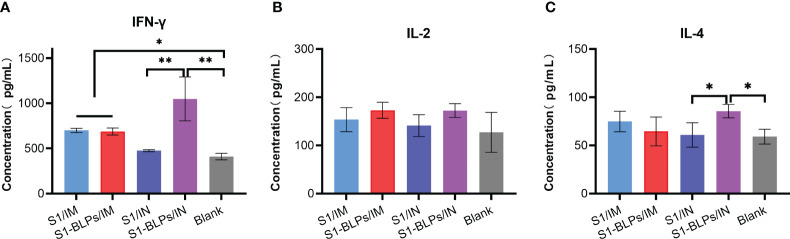
The levels of cytokines in serum by ELISA at 21 days post-immunization. **(A–C)** Concentration of IFN-γ, IL-2, IL-4 in serum, respectively. The data came from three parallel replicates of each sample. Bars show mean ± SD. **P<0.01; *P<0.05.

To further evaluate cytokine responses induced by S1-BLPs, the levels of IFN-γ, IL-2 and IL-4 in serum were analyzed by ELISA, and the results were shown in **Figure 5**. In addition to group S1/IN, serum IFN-γ levels in groups S1/IM, S1-BLPs/IM and S1-BLPs/IN were higher than that in the blank group. Furthermore, there was a stronger IFN-γ response in group S1-BLPs/IN compared to group S1-BLPs/IM and S1/IM (P<0.05), but there was no significant difference between groups S1/IN and S1/IM (**Figure 5A**). IL-2 levels were similar between the immunized groups and between the immunized and the blank group (**Figure 5B**). The level of IL-4 in group S1-BLPs/IN was higher than that in S1/IN and the blank group (*P*<0.05) (**Figure 5C**). The results indicated that S1-BLPs promoted IFN-γ response, and intranasal inoculation could significantly increase IFN-γ levels. Moreover, intranasal inoculation of S1-BLPs significantly induced IL-4 production.

## 4 Discussion

Since 2010, outbreaks of PEDV genogroup 2 (GII) have caused devastating losses in the global swine industry, especially the high mortality for newborn piglet (29–31). According to the genetic evolution analysis of the viral genome, PEDV strains around the world are divided into two groups (GI and GII). The GI consists of two subgroups (GIa and GIb), and the GII consists of three subgroups (GIIa, GIIb and GIIc) (32). The groups or subgroups of strains circulating in different countries are various, such as GIb and GIIb in the US, and GIIa, GIIb and GIIc in China (33–35). Cross-immune protection between different subgroups is low or variable (2), which brings challenges to prevent and control effectively PEDV. The S1 protein gene in this study was derived from PEDV QY2016 strain, which belongs to the GIIa subgroup and is a currently circulating strain. Therefore, the prepared S1-BLPs is expected to provide good immune protection against the current circulating strains. In addition, due to the pathogenic characteristics of PEDV, intestine mucosal immunity and the specific sIgA play a critical role in host resistance to the viral infection (36).

Because the mucosal immunity act as a crucial actor in the process of defense against pathogen infection, it is necessary to trigger mucosal immunity through various way (37). The intranasal route is one of the most direct and effective way of vaccination. Intranasal immunization can induce not only systemic specific but also specific local mucosal immune responses (38, 39). However, compared with subcutaneous and intramuscular injection of antigens, intranasal vaccination is less efficient in inducing systemic immunity (40, 41), which is consistent with the stronger serum IgG responses in the intramuscular vaccination (S1/IM and S1-BLPs/IM) groups than those in the intranasal vaccination (S1/IN and S1-BLPs/IN) groups in this study.

The routes of vaccination significantly affect mucosal immune response. In this study, intramuscular inoculation of S1 protein or S1-BLPs with adjuvant IMS1313 could induce high levels of the systemic specific IgG in mice, but could not trigger specific IgA response either systemic or mucosal immune responses. Consistent with other studies (41), our results demonstrated that the immunization route was particularly important for stimulating mucosal immunity. In addition, by comparing specific IgA levels in serum and intestinal lavage, it is found that S1-BLPs can stimulate mucosal immune response better than S1 protein. This result is similar to previous studies on influenza (42), respiratory syncytial virus (RSV) (43), and streptococcus pneumoniae bacterium-like particles (44). Moreover, Induction of mucosal immunity by S1-BLPs might be associated with the immunomodulatory effects of GEM particles as an adjuvant. According to the common mucosal immune system (CMIS), intranasal route is more practical to stimulate broad and disseminated antigen-specific mucosal and systemic immune responses (45). For the prevention and control of PEDV or other enteroviruses, the feasibility of intranasal vaccination needs further study. Most noteworthy, no specific sIgA was detected in bronchoalveolar lavage fluid of all groups, even by intranasal immunization in present study. This result is different from previous study reports on influenza mucosal BLPs vaccines (23, 42). We speculate that this result is related to the diversity of antigen characteristics and tissue tropism of PEDV. Studies have shown that the target organ of PEDV infection is the small intestine rather than the respiratory organ, and S1 protein is the key for PEDV to bind to the cell receptors (46). In addition, A combined immunization scheme (i.e., subcutaneous inoculation followed by intranasal inoculation) can induce stronger systemic and mucosal immune responses than one immunization route alone (40). Therefore, we will try different routes of combined immunization to enhance the mucosal immune effect in the future.

Furthermore, Th cell immune responses are divided into two types, Th1 response and Th2 response (47). The Th1-type immune response induces cellular immunity, while the Th2 immune response favors humoral immunity. Cytokines play a vital role in regulating immune response and maintaining the immune balance between Th1-type and Th2-type responses (48). IFN-γ, IL-2 and TNF-α/β, and IgG2a increase in Th1-type responses, while IL-4, IL-6 and IL-10, and IgG1 are elevated in Th2-type responses (47, 49). IgG2a response are associated with increased efficacy of vaccination, and more efficient at clearing virus infection (50). After intranasal administration, GEM particles, can effectively stimulate systemic and local immune responses, and enhance Th1-type immunity (25, 51). This study found that S1-BLPs significantly increase the levels of IFN-γ or IgG2a when intranasal and intramuscular administration, respectively, both of which are manifestation of Th1 type immunity. At the same time, it was found that S1-BLPs also increase the expression of IL-4 which is related to humoral immune response via intranasal inoculation. These scenarios indicate that S1-BLPs enhanced the cellular immune responses, but also stimulated humoral immune response, thereby maintaining the immune balance.

GEM particles derived from different species or strains of lactic acid bacteria display various adjuvant properties (52). Previous studies demonstrated that GEM particles from *Lactobacillus rhamnosus* CRL1505 shows stronger antiviral immune responses in porcine intestinal epithelial cells than those from Lactobacillus plantarum CRL2506 (53). Orally administered recombinant HEV capsid protein (ORF2) and GEMs from *Lactobacillus rhamnosus* strain IBLPS027 induced a more effective specific secretory IgA than that of strain CRL1505 in the gut of mice (54). Therefore, the diversity of immune-enhancing effects of lactic acid bacteria should be considered in the development of bacteria-like particle vaccines based on GEMs.

In conclusion, S1-BLPs prepared by GEM particle surface antigen display technology could induce specific systemic and intestinal mucosal immune responses in mice. Intramuscularly administered S1-BLPs significantly induced the serum specific IgG responses and increased the ratio of IgG2a/IgG1, but did not stimulate systemic and intestinal specific secretory IgA responses. Intranasally immunized S1-BLPs but not S1, could induce systemic and intestinal specific secretory IgA responses. Moreover, intranasally inoculated S1-BLPs significantly increased IFN-γ responses which is conducive to Th1-type immunity. S1-BLPs has the potential to be developed as PEDV mucosal vaccine. The next step will be to confirm whether oral inoculation of S1-BLPs can also induce the same immune effect, and to conduct *in vivo* experiments in pigs to systematically evaluate its clinical application effect.

## Data availability statement

The original contributions presented in the study are included in the article/**Supplementary Material**. Further inquiries can be directed to the corresponding author.

## Ethics statement

The animal study was approved by Hebei Agricultural University (China) Animal Welfare and Ethical Review Board. The study was conducted in accordance with the local legislation and institutional requirements.

## Author contributions

KS: Conceptualization, Data curation, Formal Analysis, Methodology, Visualization, Writing – original draft. YW: Data curation, Formal Analysis, Methodology, Visualization, Software, Writing – review & editing. CY: Data curation, Methodology, Visualization, Writing – review & editing. YZ: Data curation, Visualization, Writing – review & editing. YL: Data curation, Writing – review & editing. TL: Data curation, Software, Writing – review & editing. QS: Conceptualization, Formal Analysis, Funding acquisition, Methodology, Project administration, Resources, Supervision, Validation, Visualization, Writing – review & editing, Software.
