# Supplementary figures and images for: Intranasally inoculated bacterium-like particles displaying porcine epidemic diarrhea virus S1 protein induced intestinal mucosal immune response in mice
Source: Front Immunol. 2023 Sep 18;14:1269409. doi: 10.3389/fimmu.2023.1269409 (PMC10544335; doi:10.3389/fimmu.2023.1269409)

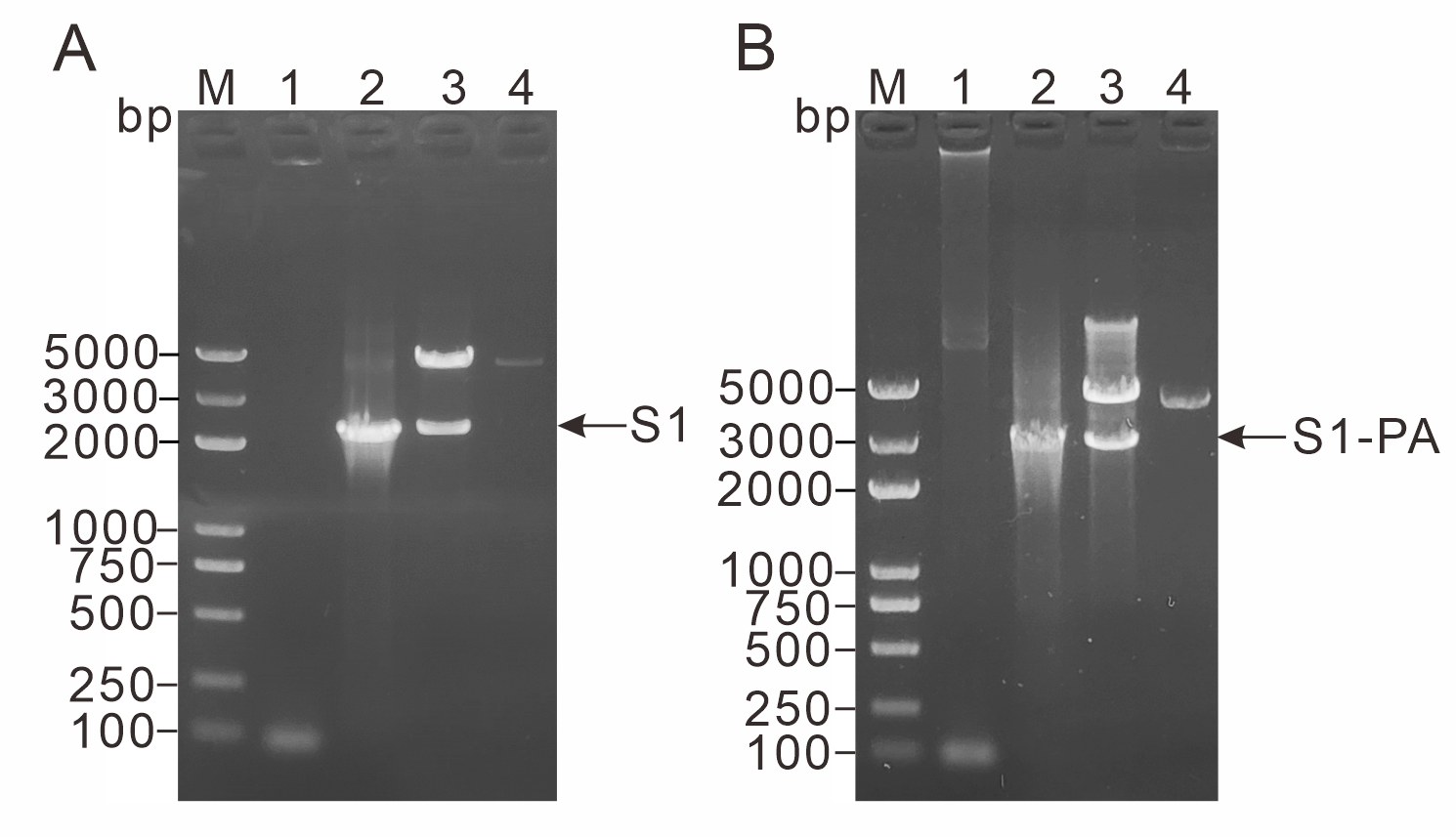

Supplement: Supplementary file 1 [file Image_1.tif]

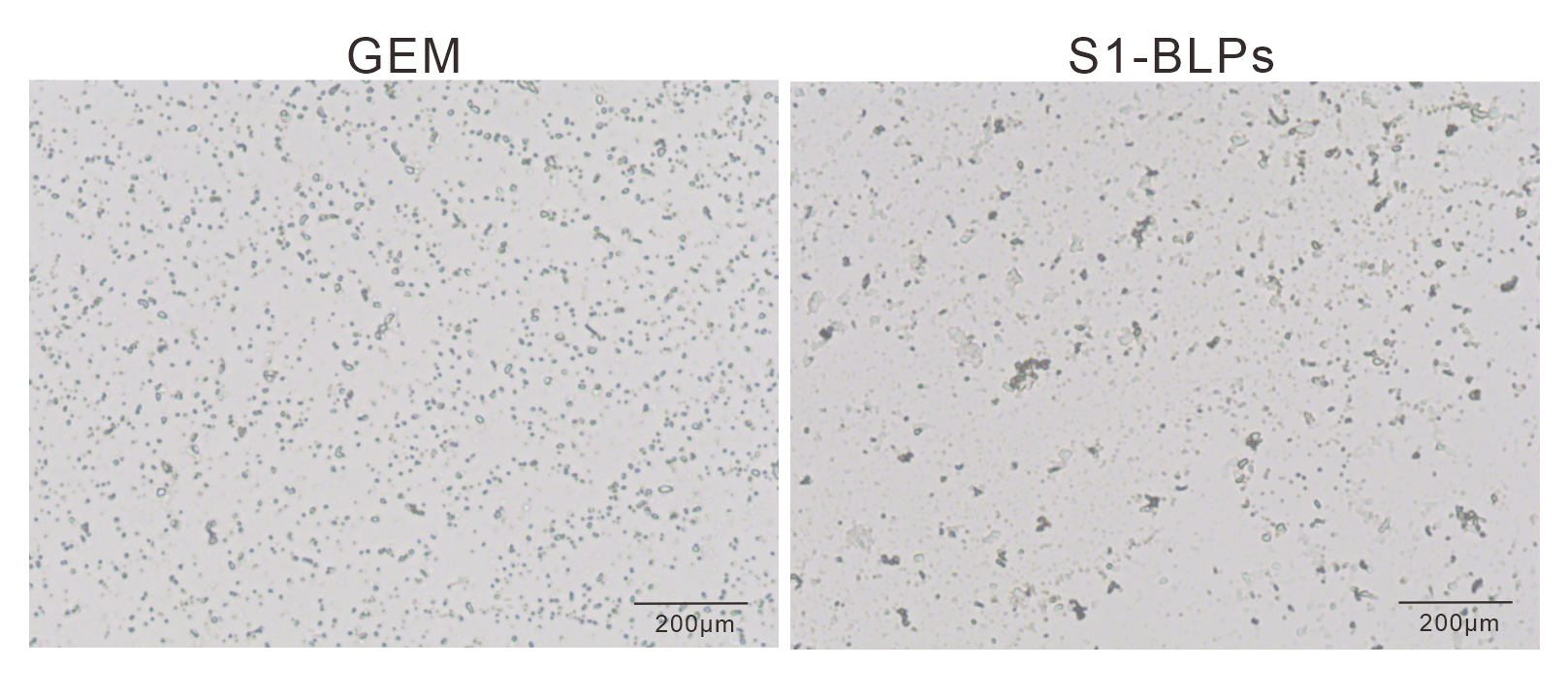

Supplement: Supplementary file 2 [file Image_2.tif]
